# Pityriasis rubra pilaris induced by topical use of imiquimod 5%^☆^

**DOI:** 10.1016/j.abd.2022.04.016

**Published:** 2023-06-30

**Authors:** Lucia Armillas-Lliteras, Maribel Iglesias-Sancho, Arcadi Altemir, Juan Antonio Moreno Romero

**Affiliations:** a Department of Dermatology, Hospital Universitari Sagrat Cor, Grupo Quirónsalud, Barcelona, Spain; b Department of Dermatology, Hospital General de Catalunya, Grupo Quirónsalud, Barcelona, Spain

Dear Editor,

Pityriasis rubra pilaris (PRP) is a rare papulosquamous inflammatory dermatosis whose pathogenesis remains unclear, it is accepted that some drugs may be involved.^1^ Four cases of PRP triggered by imiquimod have been previously reported.^2^, ^3^, ^4^, ^5^

A 67-year-old male with no relevant medical history presented multiple actinic keratosis on the chest. Imiquimod 5% cream was prescribed three times per week. During the fourth week of treatment, the patient developed painful, erythematous lesions at the site of application, so treatment with topical corticosteroid was started and imiquimod was suspended. Nonetheless, there was a worsening of the eruption, developing follicular papules with extensive coalescence, salmon colored, with superficial desquamation. It expanded with craniocaudal sense, involving the rest of the body with islands of sparing (Fig. 1). He also showed palmoplantar keratoderma (Fig. 2). He presented with flu-like symptoms such as myalgia and fever. Blood tests were within the normal range. A biopsy was performed revealing psoriasiform acanthosis, follicular plugging with parakeratosis at the edges of the follicular orifice, and marked acantholysis in multiple areas. Immunofluorescence studies were negative. Clinical-histopathological diagnosis of PRP was made. He denied any symptoms suggesting the current condition, so the etiology of the ongoing process was attributed to the treatment with Imiquimod. Oral acitretin was introduced at a dose of 50 mg per day achieving a maintained response after five months of follow-up.

**Figure 1:**
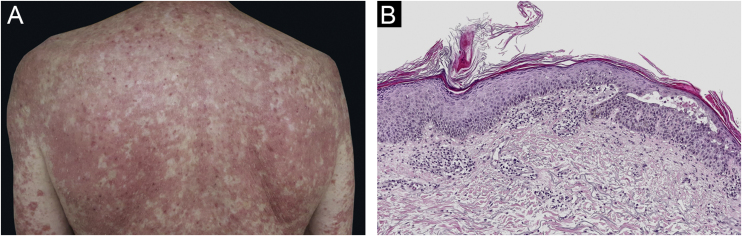
Clinical and histopathological images of the patient. (A) Salmon coloured plaques with superficial desquamation and islands of sparing. (B) Follicular plugging with parakeratosis at the edges of follicular orifice and marked acantholysis at multiples areas (Hematoxylin & eosin, ×10)

**Figure 2:**
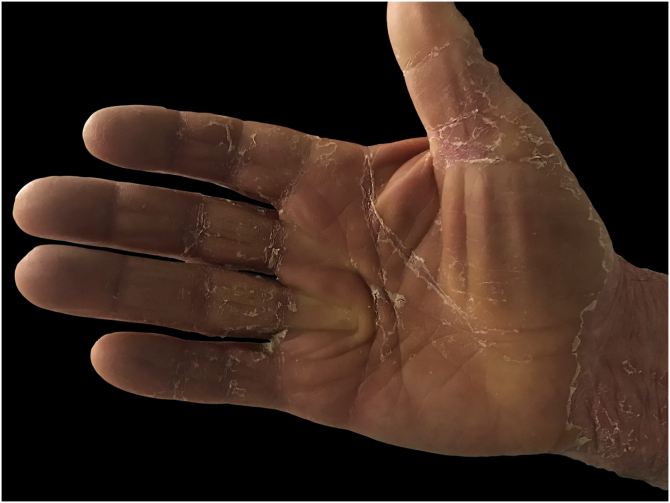
Clinical image of palmar keratoderma

PRP is a keratinization disorder, whose pathogenesis remains unclear. It has been postulated that it could be an exacerbated immune response to antigenic triggers. The T-Helper1 (Th1) pathway is activated, causing altered signaling of retinoid keratinocyte receptors, blocking the vitamin A action, and developing a keratinization disorder.^2^

Imiquimod is a topical treatment approved for many tumoral and viral diseases. It is an immune response stimulating agent, binding to Toll-Like Receptor (TLR)-7, which activates the Th1 pathway, resulting in a proinflammatory cascade, as it occurs in PRP.^3^

Four more cases of PRP induced by imiquimod with some features in common (Table 1) have been reported. It is remarkable that all of them showed acantholysis in light microscopy,^2^, ^3^, ^4^, ^5^ while it has been described in approximately only 30% of PRP biopsies in previous studies.^1^ Imiquimod has been previously reported to be involved in developing acantholytic alterations with negative direct immunofluorescence studies. It has been proposed that acantholysis could be a result of the increased levels of proinflammatory cytokines induced by imiquimod.^4^ Also, it should be pointed out that most of the patients presented systemic symptoms.^5^ Most of them showed an excellent response to conventional treatments, assuming imiquimod-induced PRP is likely to have a favorable prognosis.^2^, ^3^, ^4^, ^5^

**Table 1:** Case reports of PRP associated with topical use of imiquimod

Nº	Reference	Age/gender	History of PRP	% imiquimod	Previous dermatosis	Systemic symptoms	Acantholysis	Treatment	Time to recovery
1	Yang et al.^3^	67/M	Yes	5%	Actinic keratosis	Yes	Yes	Acitretin	6 months
2	Gómez-Moyano et al.^5^	56/M	No	5%	Superficial basal cell carcinoma	Yes	Yes	Acitretin	2 months
3	Atanaskova Mesinkovska et al.^4^	65/M	No	3.75%	Actinic keratosis	No	Yes	UVB nb	Remained with lesions
4	Leite et al.^2^	60/F	No	5%	Actinic keratosis	Yes	Yes	Methotrexate	9 months
5	a	67/M	No	5%	Actinic keratosis	Yes	Yes	Acitretin	5 months

M, male; F, female; PRP, pityriasis rubra pilaris.

a Case reported at this article.

Other inflammatory dermatoses that can be developed or exacerbated with topical use of imiquimod have been reported, such as psoriasiform eruptions, pemphigus-like lesions, erythema multiforme, subacute lupus, lichen planus, and vitiligo-like depigmentation.^2^ Thus, the systemic proinflammatory role of imiquimod seems to be reinforced.

Imiquimod, as an immune response stimulating treatment, could have a systemic effect, increasing the levels of proinflammatory cytokines and Th1 response and resulting in the developing PRP. Nevertheless, we should consider imiquimod as a useful treatment for many dermatological diseases, but we should be aware of the risk of onset and exacerbation of inflammatory dermatoses.

## Financial support

None declared.

## Authors’ contributions

Lucia Armillas-Lliteras: Substantial contributions to conception and design; acquisition of data; analysis and interpretation of data; drafting and critical review of the manuscript; approval of the final version of the manuscript; agree to be accountable for all aspects of the work.

Maribel Iglesias-Sancho: Substantial contributions to conception and design; acquisition of data; analysis and interpretation of data; critical review of the manuscript; approval of the final version of the manuscript; agree to be accountable for all aspects of the work.

Arcadi Altemir: Substantial contributions to conception and design; acquisition of data; critical review of the manuscript; approval of the final version of the manuscript; agree to be accountable for all aspects of the work.

Juan Antonio Moreno-Romero: Substantial contributions to conception and design; critical review of the manuscript; approval of the final version of the manuscript; agree to be accountable for all aspects of the work.

## Conflicts of interest

None declared.
